# Links among emotional awareness, somatic awareness and autonomic homeostatic processing

**DOI:** 10.1186/s13030-016-0059-3

**Published:** 2016-05-10

**Authors:** Kenji Kanbara, Mikihiko Fukunaga

**Affiliations:** Department of Psychosomatic Medicine, Kansai Medical University, 2-5-1, Shinmachi, Hirakata, Osaka 573-1010 Japan

**Keywords:** Emotional awareness, Alexithymia, Somatic awareness, Interoception, Interoceptive awareness, Alexisomia, Autonomic function, Homeostasis, Physiological, Psychosomatic medicine

## Abstract

Emotional awareness and somatic interoceptive awareness are essential processes for human psychosomatic health. A typical trait of lacking emotional awareness related to psychosomatic symptoms is alexithymia. In contrast, alexisomia refers to the trait of lacking somatic awareness. Links between emotional and somatic awareness and homeostatic processing are also significant for the psychosomatic health. The purpose of the present paper is to review the links among emotional awareness, somatic interoceptive awareness and autonomic homeostatic processing. On the basis of the collected evidence, the following arguments were presented^1^: (1) The main subcortical neural substrates for these processes are limbic-related systems, which are also responsible for autonomic functions for optimization of homeostatic efficiency. (2) Considerable studies have shown that autonomic activity and/or reactivity to stress correlate with both emotional and interoceptive awareness. A hypothesis was advocated about the links between the two types of awareness and autonomic function: Autonomic dysfunction, especially high sympathetic tone at baseline and/or attenuated reactivity or variability to stress, appears to be involved in disturbance of emotional and interoceptive awareness. (3) Several studies suggest that a link or a cooperative relationship exists between emotional and somatic awareness, and that somatic awareness is the more fundamental of the two types of awareness. Emotional awareness, somatic awareness and autonomic homeostatic processing generally occur in parallel or concurrently. However, some complex features of pathologies include coexistence of reduced interoceptive awareness and somatosensory amplification. The autonomic homeostatic process is fundamentally involved in emotional and somatic awareness. Investigation of these types of awareness with both neuroimaging evaluations and estimation of peripheral autonomic function are required as next steps for exploration of the relationship between awareness and human somatic states including somatic symptoms as well as general psychosomatic health.

## Background

Emotional awareness and somatic awareness are essential processes for human psychosomatic health, because disturbance of these types of awareness leads to unhealthy conditions through obstruction of homeostatic processing. Emotional/somatic awareness is the state in which individuals have access to their own emotional/somatic condition [1]. A typical trait of lacking emotional awareness related to psychosomatic symptoms is called alexithymia. In contrast, somatic awareness is physiologically based on interoception, which is defined as the homeostatic afferent neural system that represents the physiological condition of the body in humans [2–4]. Alexisomia is a term that, in contrast with alexithymia, refers to a trait of lacking somatic awareness [5]. The mechanisms that link these traits to unhealthy conditions include implicit emotional processing [6–8], disconnection between neocortical and subcortical systems [9, 10], and homeostatic inadequacy by blunt interoception [11, 12] as will be described later. In the present paper, we focus on the relationships among emotional awareness, somatic awareness, and autonomic homeostatic processing.

Patients with psychosomatic disorders often present with difficulties in awareness and expression of their emotions, or alexithymia trait [13]. The alexithymia construct was conceptualized by Nemiah, Freyberger, and Sifneos [14, 15] as a trait characterized by difficulty in identifying feelings, difficulty in describing feelings, externally oriented thinking, and a limited capacity of imagination. The alexithymia trait is involved in generation and expansion of somatic symptoms and related not only to psychosomatic disorders but also to several physical illnesses [16, 17], functional somatic disorders such as functional gastrointestinal disorders [18] or so-called “medically unexplained symptoms” [19], chronic pain [20, 21] and certain types of illness behavior [22]. Better understanding of alexithymic features for patients with psychosomatic or functional somatic disorders, in both general medical settings and specialized clinical settings, is required.

Links between the emotional process and the homeostatic physiological process are important for understanding the relationship of alexithymia to somatic symptoms or disorders. Lane conceptualized the explicit and implicit emotional processes and demonstrated that alexithymia is one of the constructs related to implicit emotional process [6–8]. Explicit emotional processing in this context means that negative emotional states such as depression, anxiety and hostility are associated with the unhealthy state or disease, while implicit emotional processing means interruption in the awareness and expression of negative emotion, which leads to unhealthy physiological conditions, typically psychosomatic disorders.

They also advocated the level of emotional awareness model [6], in which greater awareness corresponds to differentiated emotion and less awareness corresponds to undifferentiated emotion or somatic sensation. According to the concept and the model, verbal expressions are accessible for well-differentiated emotions, while undifferentiated emotions tend to lead to physiological dysfunctions or somatic symptoms through implicit emotional processing.

MacLean hypothesized for the first time that interference in connections between the limbic system and the neocortical system is the basic source of psychosomatic problems [23, 24]. Emotional awareness, in this context, is an emotional processing into awareness enabled by connection between the neocortical and subcortical systems. Alexithymia is therefore assumed to be one of the disconnection syndromes [7, 9, 10]. Disconnection between the higher-level neocortical emotional processes and subcortical emotion-generating processes contributes to dysfunctions in the autonomic nervous system (ANS) and hypothalamic–pituitary–adrenal (HPA) axis [23], and hence leads to homeostatic dysregulation and finally to disease.

### Neural substrates for emotional/somatic awareness and autonomic homeostatic processing

Neuroimaging approaches have demonstrated several neural substrates for the implicit emotional and somatic processes and their links to homeostatic regulation.　The amygdala is a core limbic structure, and many studies have demonstrated its functions in emotional processing [25–28] and emotion-related memory [29, 30]. The cingulate cortex is a paralimbic structure, and the anterior cingulate cortex (ACC) was initially thought to have general affective and emotional functions [31, 32]. It is now recognized to have two subdivisions: the dorsal and ventral portions of the ACC. Although the dorsal ACC (dACC) has generally been thought to have primarily cognitive functions [33, 34], the dACC actually plays considerable roles in emotional processing [35, 36]. Moreover, several bodies of evidence using emotional awareness scales [37] for example suggest the dACC is involved in emotional awareness or expression of emotion [38, 39]. Whereas, the ventral ACC mainly connects to the amygdala, hypothalamus and insula [33, 40], it has outflow to autonomic nervous and endocrine systems, and plays a role in regulation of emotional responses with respect to limbic systems [39, 41, 42]. Overall, the amygdala is associated primarily with unconscious or implicit emotional processing, while the cingulate cortex is associated predominantly with conscious processing of emotion [43, 44].

On the other hand, the insula is a cortical area closely connected with the limbic system, which receives somatosensory signals [45]. The insula is particularly involved in interoception or interoceptive awareness [2, 3, 46]. Interoceptive stimuli include thirst, dyspnea, sensual touch, coolness, warmth, heartbeat, and so on [4]. Interoceptive representations in neural substrates are primarily via the anterior insula [4, 46], which has a fundamental role in all subjective feelings from the body [4], while the ACC is responsible for autonomic alteration along with interoception [47]. The basic purpose of interoceptive awareness is to optimize homeostatic efficiency [11]. Disturbed interoceptive awareness therefore could lead to an unhealthy condition through homeostatic insufficiency. The relationship between interoceptive and emotional awareness has been studied and several lines of evidence to be described later have suggested a cooperative relationship between the two.

Most of these neural substrates for implicit emotional processing and interoceptive awareness are also known to be responsible for the ANS and HPA axis functions, which are major homeostatic regulatory systems or allostatic systems [48, 49]. Allostasis is the process of achieving stability through physiological or behavioral change; the set point of the stability is not static but dynamic in allostatic systems [48]. Alteration of the set point could lead to disease; this constitutes one explanation for the psychosomatic process. Hence, the concept is important for psychosomatic health.

Damasio advocated a neural model, in which the neural distinctions among primary emotion, secondary emotion, and feeling are demonstrated [50]. Primary emotions are the result of an implicit subconscious emotional process that occurs as a first response to a situation, and are closely related to adaptive and survival processes; in contrast, secondary emotions are the outcome of a process that occurs after the primary emotions, and represent social and higher-level processes with the experience of consciously processed emotion. The primary emotional response is considered the phylogenetically older behavioral and a physiological expression of the emotional response [7]. Meanwhile, interoception is a homeostatic afferent pathway and sensory aspects of homeostasis that represent the physiological condition of the body [2]. Thus, the implicit emotional and interoceptive processes have a basal relationship with the homeostatic process through ANS and HPA axis.

Considering these theories and arguments together, both emotional awareness and somatic interoceptive awareness are essential processes for human psychosomatic health. The homeostatic physiological process—that is, autonomic and HPA functions, or its response to environment or stress—is intimately linked to emotional and interoceptive awareness; therefore, this process is one of the most important clues to investigate both types of awareness. The ANS is key for achieving the appropriate modification of physiological parameters, although the hormonal system is activated simultaneously [51]. Additionally, the autonomic indexes are easy to measure and easy to apply in psychophysiological approaches such as biofeedback to psychosomatic patients. We have therefore directed attention to autonomic response to stress as a homeostatic marker. Here, the purpose of the present paper is to review evidence of the links among emotional awareness, somatic interoceptive awareness and autonomic homeostatic processing.

### Emotional awareness and autonomic homeostatic processing

Many studies have addressed autonomic characteristics in response to stress tasks or various stimuli in alexithymic individuals. The findings vary by experimental condition and have some inconsistencies [52]. Some studies indicate a hyperarousal model of alexithymia, in which higher autonomic responses are observed in alexithymic individuals [53, 54], whereas considerable studies support a hypoarousal model of alexithymia, in which attenuated autonomic reactivity inhibits the perception of emotional signals [55–57]. These inconsistencies are probably due in part to differences in stimuli (e.g., tasks involving mental arithmetic, affective picture viewing, or social speech stress) and differences in physiological measurements (e.g., heart rate or other cardiovascular marker, electrodermal activity or skin conductance level, or muscle tension).

Although results have diverged in some areas, two findings are mostly consistent. (1) Autonomic tone at baseline, as measured by electrodermal activities such as skin conductance, is higher in alexithymic than in non-alexithymic individuals, whereas responses to stress (measured as change scores from baseline) are relatively low [53, 54, 58–60]. (2) In contrast, baseline autonomic states, measured by heart rates, are unrelated to alexithymic levels, while the responses to stress differ according to stimulus types [58, 59, 61, 62].

**Fig. 1:**
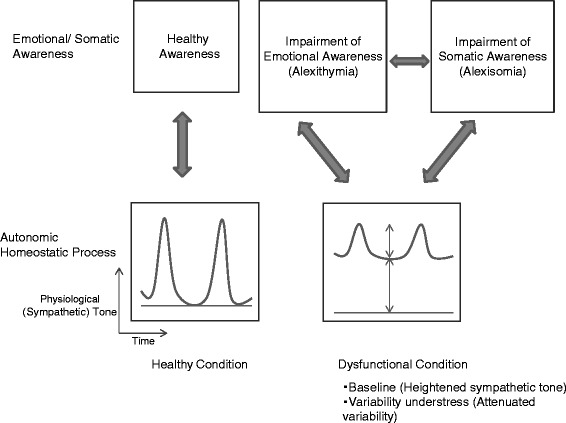
Hypothesis of links among emotional awareness, somatic awareness and autonomic homeostatic processing. Autonomic dysfunction, especially heightened sympathetic tone at baseline and/or impaired variability or reactivity to stress, is involved in the disturbance of emotional and interoceptive awareness. In a dysfunctional condition of the autonomic nervous system, low variability of physiological tone contributes to disturbance of proper perception in emotional/somatic inputs, and the disturbance leads to the impairment of emotional and/or somatic awareness

Electrodermal activities are known to be an index of autonomic tone related to emotional arousal that is mainly controlled by the sympathetic system [63], while cardiac activities are regulated by both sympathetic and vagal functions. Electrodermal activities are also known to follow the “law of initial value,” which means that high baseline levels limit the amount of change that can be produced by stimuli [64]. Another study using cortisol levels suggested increased basal HPA activity in alexithymic subjects [65]. The HPA axis and sympathetic-adrenal-medullary systems are major stress-responsive systems that work in alliance and react concurrently in most cases with stressful situations [66]. On the basis of these reviews, it is plausible that heightened resting sympathetic tone and/or attenuated reactivity to stress, or variability, are involved in the disturbance of proper perception of emotional inputs in a majority of alexithymic individuals (Fig. 1).

Additionally, several studies [65, 67] have reported varying autonomic or HPA reactivity results with different subscales of the 20-item Toronto Alexithymia Scale (TAS-20) [68, 69]. For example, Pollatos et al. demonstrated that only the alexithymia facet “difficulty in describing feelings” was associated with smaller electrodermal responses [67]. Bermond and Vorst presented evidence that the alexithymia trait has both affective and cognitive dimensions that are orthogonal to one another, and proposed different physiological features in these dimensional categories [70, 71]. Therefore, such heterogeneity of alexithymia construct should also be considered as a source of the variety in outcomes of the studies described earlier.

Our previous studies in patients with psychosomatic or functional somatic symptoms, who generally have been thought to have difficulties in emotional and/or somatic awareness, showed overall hyporeactivity to a mental arithmetic stress task in several physiological measurements including skin conductance level, skin temperature, and blood volume pulse amplitude [72, 73]. A subsequent study with a larger sample identified two clusters of psychophysiological stress response patterns: a majority cluster whose members have low stress response patterns and another cluster with high or even response patterns [74]. Another study suggested that functional somatic syndrome patients with difficulty in identifying feelings had higher sympathetic tone at pre-stress baseline, measured by salivary amylase [75, 76]. Moreover, the alexithymic trait and resting sympathetic tone correlated positively in healthy controls [75].

Considering these findings and aforementioned reviews together, several dysfunctional states in ANS are involved in the disturbance of emotional awareness; especially, high sympathetic tone at baseline and/or impaired autonomic variability affect emotional awareness in a number of alexithymic individuals (Fig. 1). Whereas, the alexithymia construct is heterogeneous, and one or more other mechanisms could also affect emotional awareness in other types of alexithymia.

### Emotional awareness and feedback model through vagal function

Several studies have indicated that the function of the medial prefrontal cortex, which has a strong connection to limbic structures such as the amygdala, is correlated positively with autonomic vagal activity measured by the vagal component of heart rate variability [77–80]. The ACC, which is anatomically adjacent to the medial prefrontal cortex, has also been shown to have a positive correlation with vagal function [81].

The medial prefrontal cortex and the ACC have a role of conscious processing of emotion as described previously [7, 8]. Conscious processing or conscious awareness of emotion is generally accepted clinically as an important process for emotional self-regulation [82]. Lane et al. argued that conscious processing of emotion requires the transmission of subcortical affective information to the cerebral cortex − medial prefrontal cortex and ACC in this context [80]. The processing also requires top-down feedback from cortical to subcortical function [43, 80]. They considered the positive correlation of the cortical activity with vagal function described previously as evidences of top-down feedback [7, 80].

**Fig. 2:**
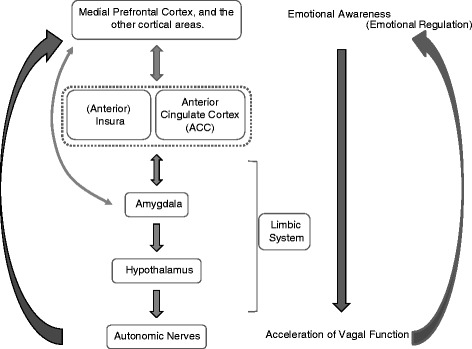
Negative feedback model in emotional awareness and vagal function, and proposed neural pathway. Conscious processing of emotion (emotional awareness) has a negative feedback loop through the vagal function [6, 7, 83]. Emotional experience is consciously processed into awareness, and vagal tone is accelerated, after which emotional arousal is regulated. The neural pathway for the feedback process is assumed to involve cortical areas such as ventrolateral prefrontal cortex and especially medial prefrontal cortex, insula, limbic system structures such as anterior cingulate cortex (ACC), amygdala and hypothalamus, and the autonomic nervous system. The anterior insula and ACC have a functional relationship for the emotional processing (see also in the text). Various other pathways, a more direct connection for example, could be included in the processing, but this Figure was simplified for a brief explanation of the model

Based on the existing evidence and its interpretations, the conscious processing of emotion is assumed to have a negative feedback loop through vagal function [6, 83]; that is, when emotional experience is consciously processed into awareness, vagal tone is accelerated, after which emotional arousal is regulated [7]. In other words, the medial prefrontal cortex and ACC have a tonic inhibitory effect through vagal activity when these areas are activated in connection with conscious emotional experience. Vagal tone has been considered to have a function of regulating emotional responses [84], or selecting an optimal response and inhibiting less optimal ones [80] (Fig. 2).

Research suggests that the neural pathway for the feedback process involves cortical areas, from the ventrolateral prefrontal cortex and especially the medial prefrontal cortex; through limbic system structures such as the cingulate cortex (especially the ACC), amygdala and hypothalamus; to the ANS [7, 47, 83, 85] (Fig. 2). The insula is also involved in this pathway in addition to being a representative center for interoception as described before. The anterior insula and ACC have a close functional relationship and the two areas conjointly bear an important role in emotional processing [86].

If conscious processing is disturbed, vagal tone would not be accelerated, and the resting sympathetic tone would be easily activated. This negative feedback model suggests a mechanism of the relationship between the autonomic function and emotional awareness, and supports the hypothesis shown in Fig. 1.

### Interoceptive awareness and autonomic homeostatic processing

Autonomic activity or reactivity to stress is involved in interoceptive awareness as well as emotional awareness. Some studies have suggested that individual difference in sympathetic cardiac activity affect interoceptive sensitivity to heartbeats [87, 88]. Several subsequent studies with heartbeat perception tasks [89, 90] have indicated that cardiac interoceptive awareness is affected by autonomic reactivity to some tasks [91–93]. These studies suggested that interoceptive awareness is positively related to the autonomic response to such tasks.

Herbert in particular established that cardiac awareness was associated with greater sympathetic reactivity during mental stress and greater vagal reactivity during emotional picture viewing [93]. Both sympathetic and vagal activities could contribute to cardiac awareness [93]. Autonomic function or reactivity therefore affects not only emotional awareness but also somatic interoceptive awareness.

### Interoceptive awareness and emotional awareness

James first described the idea that emotional experience necessarily included perception of a somatic response [94, 95], and Lange proposed a similar concept [96]. Since then, historical debates on the relationship between emotional experience and somatic change have continued and have influenced theories of interaction between emotions and the body.

Considerable studies with a heartbeat perception task as an interoceptive index have demonstrated that subjective emotional experience relates positively to interoceptive sensitivity [46, 97, 98]. Consistent with these findings, evidence exists that alexithymic individuals have less interoceptive awareness [99]. Several studies using electroencephalogram (EEG) recordings also suggest interoception correlates positively with emotion processing, measured as P300 event-related potentials, for example [100–102].

Studies using heartbeat-evoked brain potential, which is observed by EEG promptly after the R-wave of the electrocardiogram over the somatosensory cortex and the frontal/prefrontal cortex, have also examined cortical processing of signals from cardiovascular activities [103, 104]. The heartbeat-evoked potential could be a plausible neurophysiological marker of cardiac interoceptive awareness [105] and has a potential significance for exploration of the relationship between cardiac interoception and cortical awareness processes. Heartbeat-evoked potentials have been linked to empathy [106], suggesting a correlation between cardiac awareness and emotional processing.

The anterior insula, a core substrate for interoception, is also activated by emotional processing [107, 108]. Neuroimaging studies suggested that the common activated areas for interoceptive awareness and emotional experience were the right anterior insular cortex and ventromedial prefrontal cortex [109, 110]. These findings suggest the close neurophysiological correlation of emotional and somatic interoceptive awareness. Additionally, difficulties in emotional awareness, i.e., alexithymia, are in most cases clinically associated with difficulties in somatic awareness, or alexisomia [5].

In contrast, several studies have suggested activation of some somatosensory systems in alexithymic individuals. For example, visceral sensitivity to stimulation is associated with alexithymia [111]. Alexithymic persons have exhibited higher activation in the pain matrix area of brain [112]. Women with alexithymia have demonstrated higher activation of sensory and motor cortex, compared with controls [113]. These findings are consistent with the psychosomatic theory in which alexithymic patients tend to express their conflicts of emotion somatically rather than linguistically.

It is seemingly contradictory that emotional and somatic interoceptive awareness positively correlate, while impairment of emotional awareness leads to somatosensory amplification. Moriguchi and Komaki elucidated this argument by demonstrating the limitation of TAS-20 as a self-report measure and the heterogeneity of alexithymia [114]. As mentioned before, Bermond and Vorst hypothesized subtypes of alexithymia by dimensional model: one characterized by attenuation of physiological arousal, and another marked by intact physiological arousal [70]. This typology could partially explain the apparent contradiction.

Another explanation is suggested by the difference between interoceptive awareness and somatosensory amplification [115]. For example, a number of patients with chronic pain, who have less emotional and interoceptive awareness, experience persistence of a specific type of pain, such as myalgia of some part of the body, with somatosensory amplification. Barsky et al., who advocated the concept of somatosensory amplification, demonstrated that patients with hypochondria had high somatosensory amplification but were not good at cardiac awareness [116, 117]. Moreover, Mailloux and Brener directly examined the relationship between somatosensory amplification and cardiac interoceptive awareness [118]. They showed greater somatosensory amplification in poor heartbeat detectors than in good detectors, and suggested that somatosensory amplification is a “cognitive bias” and does not reflect heightened somatic awareness.

Interoceptive awareness and somatosensory amplification are therefore not on the same axis. At its core, somatosensory amplification is a condition in which sensation of a somatic state is higher than the assumed actual state, whereas interoception is an “accurate” sensation of a somatic state; therefore, reduced interoception could mean either a hyper- or hyposensitive condition. Individuals with less interoceptive or emotional awareness may not perceive their own somatic or emotional state properly and may exhibit somatosensory amplification.

According to psychosomatic theory, the amplification comes not only from cognitive bias but also from physiological bias. Kano and Fukudo reported in their recent review that alexithymic individuals showed reduced responses in limbic areas in their cognitive processing, but amplified responses in more primitive processing, e.g., hypersensitivity to visceral pain [119]. Somatosensory amplification in more primitive processes may occur typically in psychosomatic patients who have less emotional and interoceptive awareness.

## Discussion

Several key points were presented in this paper^1^. First, both emotional and somatic interoceptive awareness are important for psychosomatic health. The main subcortical neural substrates for these processes are limbic-related systems: the amygdala, cingulate and insula, which are also responsible for autonomic and HPA axis functions for optimization of homeostatic efficiency. Second, considerable studies show that autonomic activity and/or reactivity to stress correlates with both emotional and interoceptive awareness. The hypothesis that autonomic dysfunction is involved in the disturbance of emotional and interoceptive awareness has been advocated (Fig. 1). Third, several sources of evidence suggest links or a cooperative relationship between emotional and somatic awareness. However, impairment of emotional or somatic awareness often accompanies somatosensory amplifications in a specific area or system, typically in psychosomatic patients.

The first point suggests links between emotional/interoceptive awareness and psychosomatic health through homeostatic or allostatic process. The so-called emotional awareness or somatic awareness approach, which is assumed to accelerate emotional or somatic awareness, could also accelerate the homeostatic process and lead to psychosomatic health. One of the key mechanisms for psychosomatic health is links between limbic-related systems and higher cortical systems. The emotional/somatic awareness approaches are assumed to accelerate these links.

### Awareness and autonomic function: Clinical implications

Regarding the second point, in which both emotional and somatic awareness correlate with autonomic function, increased awareness could improve autonomic function and consequently autonomic-related symptoms; or reciprocally, the improvement of autonomic function could facilitate awareness. A number of neuroimaging studies have demonstrated that putting emotional feeling into words, i.e., affect labeling, changed the response to stimuli of the amygdala and other limbic structures [85, 120]. These results suggest that conscious awareness and linguistic processing of emotion effect alterations in autonomic function. Although it is inconclusive whether emotional awareness or linguistic processing actually effects the alteration of limbic function, the linguistic process necessarily involves conscious awareness, and both processes could improve autonomic function. The aforementioned negative feedback model, in which conscious awareness of emotion accelerates vagal function [6, 83] (Fig. 2) supports these arguments.

Interoceptive awareness also could improve autonomic function in a way suggested by the second and third points and arguments in the last two sections. In the context of the negative feedback model, interoceptive awareness probably accelerates vagal function, which leads to the reduction of symptoms. Actually, Schaefer et al. reported that improving interoceptive awareness reduced symptom distress in patients with somatoform disorders or medically unexplained symptoms [121, 122]. Further empirical verification of the relationship between interoceptive awareness and the vagal feedback process is required.

On the basis of this review, the hypothesis presented in Fig. 1, and our previous studies, in which psychosomatic patients had low autonomic responsiveness, we conclude that autonomic responsiveness is involved in one of the most important processes for emotional/somatic awareness upon psychosomatic health. Our previous study also suggested co-occurrence of low variability in subjective feelings of tension and attenuated autonomic response [72]. Low variability in autonomic function and subjective feeling probably contributes to disturbance of emotional and/or interoceptive awareness through less discriminative sensation. Excessive variability also may contribute to this disturbance. We therefore tentatively consider variability in autonomic function as one of the most important contributing factors for emotional/somatic awareness although this hypothesis needs further verification study.

The autonomic dysfunctional condition interacting with the impairment of emotional and/or somatic awareness seen in Fig. 1 leads to unhealthy psychosomatic conditions through insufficient homeostatic or allostatic process along with autonomic dysfunction. The condition should be ameliorated by improvement in emotional/somatic awareness.

### Alexithymia and alexisomia

Regarding the third point, either the emotional or somatic form of awareness could facilitate the other. The concept of “response system coherence” [123–125], that is, the idea that emotions organize and synchronize different response systems (e.g., behavioral and physiological) [126], can explain the cooperative relationship between emotional and somatic awareness. Sze et al. reported that the coherence between subjective emotion and cardiac awareness was greater in those who had specialized training in promotion of somatic awareness than did controls [126]. Our arguments are consistent with the clinically accepted process that “body-oriented” approaches such as yoga, biofeedback, and certain kinds of body psychotherapies encourage the emotional process of awareness, i.e., improvement of the alexithymic trait.

Ikemi, a founder of psychosomatic medicine in Japan, first described the concept of alexisomia in association with alexithymia in the early 1980s as clinical characteristics of difficulties in awareness or expression of somatic feeling/sensations from an Eastern point of view [5, 127]. Mind and body are considered as one harmonic entity in the Eastern view, but as dualistic entities in the Western view. The concept of alexisomia was developed on the basis of the Eastern conceptualization, but the concept has not yet been explored extensively. Meanwhile, the mechanism of interoceptive awareness has been investigated in the context of neurophysiological studies as described in the present paper. Impairment of interoceptive awareness appears to be similar to alexisomia, although a conclusive decision about whether these states are exactly the same cannot yet be made.

Regarding the relationship between alexisomia and alexithymia, Moriguchi and Komaki addressed this topic with reference to reviews of neuroimaging studies and concluded that somatic awareness is the basis of emotional awareness because bodily states, including autonomic and hormonal status, are fundamentally involved in basic affective states [114]. Damasio advocated the somatic marker hypothesis, wherein emotional processes lead behavior, especially the choice of a proper action or decision, and demonstrated the fundamental role of the physiological state in emotional processing [50, 128].

Therefore, although emotional awareness, somatic awareness and autonomic processing are generally parallel or concurrent processes, the aforementioned arguments suggest that somatic awareness is fundamental for emotional awareness, and that the autonomic homeostatic process has a more fundamental involvement in somatic and emotional awareness. However, psychosomatic patients with alexithymia/alexisomia have complex pathologies, which include coexistence of impairment of interoceptive awareness and somatosensory amplification, as previously described. This complexity might be associated with the multiple pathologies seen in patients not only with psychosomatic disorder but also with chronic pain or functional somatic syndromes.

Another source of complexity is that the process of awareness is involved in various levels of processing such as peripheral/sensory, intermediate/limbic related and central/cognitive systems. Alexisomia includes impairments of not only peripheral processes but also cognitive or even higher-level processes [5]. One of the challenges and difficulties for investigation of mechanisms of alexisomia is that this multilevel construct applies here as well as in alexithymia. Further clinical and physiological investigations that consider such multilevel properties are needed for better understanding of the process of alexisomia and interoceptive awareness.

### Self and awareness

Finally, any arguments about awareness, which can be seen as a change in the subjective–objective relationship, must include some mention of the notion of “self”, because subjective feelings necessarily require a self that experiences the feelings. Craig stated that to have awareness is to know that one exists [4] and addressed the neural model for integrative representations of all feelings from the body at any moment as “the sentient self” [11]. Damasio posited that the self is a repeatedly reconstructed biological state, called “neural self” [129]. Thus, the self is inseparable from the somatic and emotional feelings or senses and awareness is a state of linkage between the self and the senses. This theme is extremely profound and beyond the scope of our paper, but clinically, impairment of emotional/somatic awareness likely relates to disestablishment of the self. We may therefore have to consider the need for reestablishment of the self in our approach to patients with difficulties in these types of awareness or alexithymia/alexisomia, especially children or persons with developmental disorders.

## Conclusions

This paper reviewed links between emotional awareness, somatic awareness including interoception, and autonomic homeostatic processing, and advocated a hypothesis about the links between the two types of awareness and autonomic function. Autonomic homeostatic processing has a fundamental involvement in both emotional and somatic awareness. Investigation of these types of awareness, using both neuroimaging evaluations and estimation of peripheral autonomic function, are required next steps for exploration of their relationship to somatic symptoms, and to the more general issue of human psychosomatic health.

## Endnote

^1^For readability, the key points of this manuscript are indicated as (1), (2), and (3) in the abstract. The numbers correspond to points labeled as “first,” “second,” and “third” in the Discussion section. The first point also relates to the context in the Background and to the subsection titled “Neural substrates for emotional/somatic awareness and autonomic homeostatic processing.” The second point corresponds to the following subsections in the main text: “Emotional awareness and autonomic homeostatic processing,” “Emotional awareness and feedback model through vagal function,” and “Interoceptive awareness and autonomic homeostatic processing.” The third point corresponds to the subsection titled “Interoceptive awareness and emotional awareness.”

## Abbreviations

FSS: functional somatic syndrome
ANS: autonomic nervous system
HPA (axis): hypothalamic pituitary adrenal (axis)
(d) ACC: (dorsal) anterior cingulate cortex
TAS-20: 20-item Toronto Alexithymia Scale
EEG: electroencephalogram
